# Lateral Supravesical Hernia Successfully Treated With the Totally Extraperitoneal (TEP) Approach With Adjunctive Intra-abdominal Observation: A Case Report

**DOI:** 10.7759/cureus.89037

**Published:** 2025-07-30

**Authors:** Masato Hayashi, Naoki Horikawa

**Affiliations:** 1 Surgery, Takaoka City Hospital, Toyama, JPN

**Keywords:** abdominal observation, bladder injury, supravesical hernia, totally extraperitoneal repair (tep), transabdominal preperitoneal (tapp)

## Abstract

Lateral supravesical hernias are an exceptionally rare subtype of external supravesical hernias that protrude laterally to the urinary bladder through the supravesical fossa. Due to their atypical location and nonspecific clinical presentation, they are often preoperatively misdiagnosed. We report the case of an 81-year-old male who presented with right lower quadrant discomfort and a reducible inguinal bulge. Preoperative CT revealed a hernia sac, but the precise anatomical origin was unclear. Laparoscopic observation, initially performed for a transabdominal preperitoneal (TAPP) approach, identified the hernia orifice lateral to the bladder, prompting a change in strategy to a totally extraperitoneal (TEP) repair to avoid bladder injury. The hernia was successfully reduced from the preperitoneal space, and mesh was placed without peritoneal violation. The postoperative course was uneventful, and the patient remained symptom-free with no recurrence at the 12-month follow-up. This report demonstrates that intra-abdominal observation can aid in the accurate diagnosis of atypical hernias and that a TEP approach offers a safe and effective treatment method, particularly when bladder proximity poses a surgical risk.

## Introduction

Supravesical hernias are rare types of hernias situated in the supravesical fossa, located medial to the medial umbilical plica, lateral to the urachus, and on the cranial side of the iliopubic tract, pubis, and bladder [1]. The first case of external supravesical hernia was reported by Cooper et al. in 1804 [2]. Recently, Supravesical hernias have accounted for approximately 3.4% of all inguinal hernias [3]. They are categorized as medial or lateral based on whether the herniation occurs within the peritoneal cavity or through the abdominal wall, respectively. Lateral supravesical hernias, a subset of external supravesical hernias, are exceedingly rare and can present diagnostic challenges preoperatively due to their nonspecific clinical and radiological findings [4,5].

While inguinal hernias are typically diagnosed through physical examination and imaging, atypical presentations, such as supravesical hernias, are frequently misdiagnosed or only correctly identified intraoperatively [6]. Clinically, supravesical hernia may mimic direct or indirect inguinal hernias, femoral hernias, Spigelian hernias, or even obturator hernias. External supravesical hernia often presents as inguinal bulges medial to the inferior epigastric vessels, leading to frequent misclassification as true inguinal hernias [7]. Abdominal CT is the most valuable preoperative tool; suggestive findings include clustered or dilated bowel loops anterior or adjacent to the bladder and mesenteric convergence in an atypical region [8]. However, preoperative diagnosis remains uncommon, with most supravesical hernias identified intraoperatively [4]. Supravesical hernias are associated with a high risk of small bowel incarceration and strangulation, with emergent bowel resection required in approximately 20% of cases [4,8].

Minimally invasive techniques, such as the transabdominal preperitoneal (TAPP) and totally extraperitoneal (TEP) approaches, have become standard in hernia repair. The diagnosis of lateral supravesical hernias is expected to increase with the growing use of laparoscopic surgery [4]. However, each approach carries distinct advantages and risks depending on the hernia type and anatomical complexity [9]. We present a rare case of lateral supravesical hernia that was accurately diagnosed and safely repaired using the TEP approach with adjunctive intra-abdominal observation, thereby avoiding potential complications such as bladder injury.

## Case presentation

**Figure 1:**
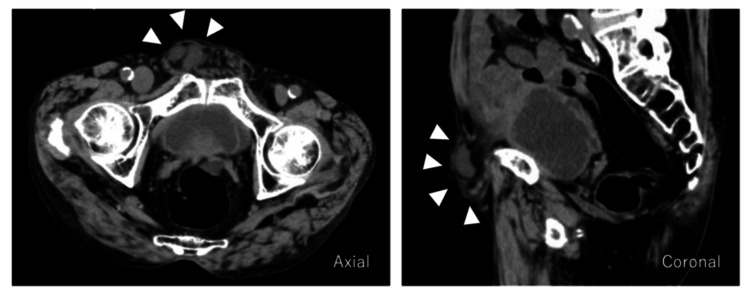
Preoperative plain abdominal CT (groin decompression prone positin) There was mild prolapse of fat tissue and a small amount of fluid in the right groin area (arrowhead) CT: computed tomography

An 81-year-old male presented with right lower quadrant discomfort and a gradually enlarging inguinal bulge. Physical examination revealed a reducible mass in the right groin area. Preoperative CT demonstrated a hernia sac in the inguinal region, but its precise anatomical origin was unclear (Figure 1).

**Figure 2:**
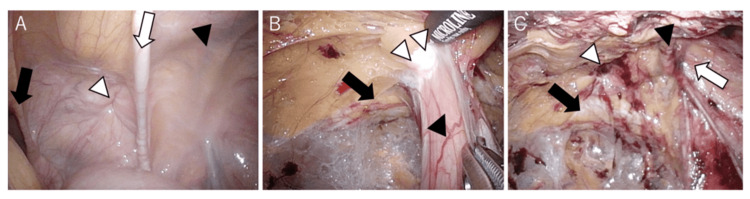
Laparoscopic surgical findings (A) A hernia orifice was found inside the right medial umbilical fold; black arrow: median umbilical fold; white arrow: right medial umbilical fold; white arrowhead: supravesical hernia; black arrowhead: inferior epigastric artery. (B) Hernia sac dissection findings using the TEP approach; black arrow: Cooper’s ligament; white arrowhead: pseudo sac; black arrowhead: prolapsed bladder. (C) The hernia orifice was dissected all around; black arrow: Cooper’s ligament; white arrow: right inguinal ring; white arrowhead: hernia orifice; black arrowhead: inferior epigastric artery TEP: totally extraperitoneal

The patient was initially scheduled for the TAPP approach. Intra-abdominal observation revealed that the hernia orifice was located lateral to the urinary bladder (Figure 2A), raising concerns about potential bladder injury if standard TAPP dissection were continued. Consequently, the surgical team elected to convert to the TEP approach. The hernia sac was successfully identified and reduced from the preperitoneal space without peritoneal violation (Figures 2B, 2C). A standard polypropylene mesh was then placed to reinforce the myopectineal orifice (MPO). No intraoperative complications occurred. The patient's postoperative course was uneventful, and he was discharged on postoperative day two. At the 12-month follow-up, there was no evidence of recurrence or chronic pain.

## Discussion

Lateral supravesical hernia is a rare form of external supravesical hernia, with only a few cases reported in the literature [4,9,10]. Clinical symptoms of a medial supravesical hernia include severe abdominal pain, intestinal obstruction symptoms such as nausea and vomiting, frequent urination, and dysuria. In contrast, lateral supravesical hernias often present with inguinal bulges, lower abdominal pain, and pain in the medial thigh [11]. We conducted a literature search in the Journal of Medical Sciences and PubMed and found 29 reported cases (including this case) of lateral supravesical hernia between February 1976 and June 2025 (keyword: "lateral or external supravesical hernia"). Preoperative CT was performed in 18 of these cases; however, only one was accurately diagnosed. The TAPP procedure was performed in most cases, and only three cases, including this case, were repaired using the TEP procedure. The hernia sac typically protrudes laterally to the urinary bladder, and its identification can be difficult both clinically and radiologically. Although CT may provide suggestive findings, definitive diagnosis often requires direct visualization during surgery [5,12].

**Figure 3:**
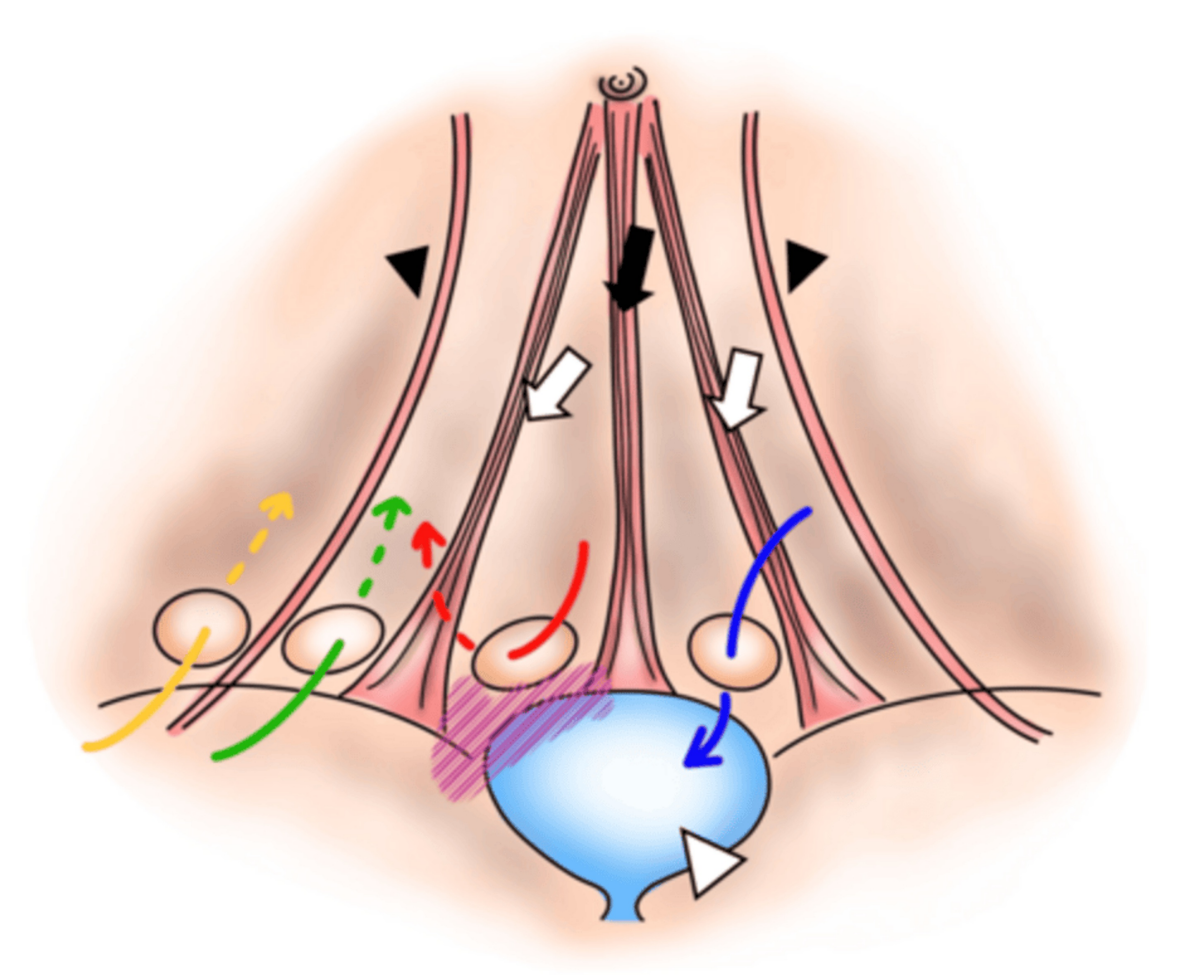
Schematic diagram of inguinal hernia Anatomical landmarks: black arrow: median umbilical fold; white arrow: right/left medial umbilical fold; black arrowhead: right/left inferior epigastric artery; white arrowhead: bladder. Hernia orifice: blue arrow: medial supravesical hernia; red arrow: lateral supravesical hernia; green arrow: direct inguinal hernia; yellow arrow: indirect inguinal hernia; purple shaded line: areas close to the bladder

In our case, the initial laparoscopic inspection raised concerns regarding the hernia's proximity to the bladder. Figure 3 illustrates a schematic diagram of an inguinal hernia. The purple shaded area is where the hernia orifice is close to the bladder, indicating a high risk of injury. This anatomical relationship makes the TAPP approach potentially hazardous due to the risk of bladder injury during medial dissection after peritoneal incision in the TAPP approach. Kameyama et al. [13] state that since the TEP approach involves dissecting from the preperitoneal cavity, the bladder must be dissected dorsally before treating the hernia sac, and that there is a lower risk of bladder damage during surgery than with the TAPP method, which requires incisions such as a peritoneal incision. Therefore, we opted for a TEP approach, which allowed us to avoid entry into the peritoneal cavity and to safely dissect the preperitoneal space [14].

It cannot be definitively stated that a bladder injury would have occurred if the TAPP approach had been continued. On the other hand, if the hernia had been repaired only with the TEP approach, it might have been misidentified as a typical direct inguinal hernia. Importantly, adjunctive intra-abdominal observation was found to be useful for making an accurate diagnosis. This report demonstrates that the combination of TEP and intra-abdominal observation can be a useful strategy in cases of atypical hernia, resulting in both a clearer diagnosis and a safer course of treatment [6,15].

We believe that our approach highlights the flexibility of laparoscopic hernia repair techniques and underscores the importance of tailoring the surgical strategy to the individual anatomical presentation. While TEP is generally considered to be more difficult in terms of recognizing anatomical landmarks than TAPP, the strategic use of intra-abdominal observation can enhance diagnostic accuracy without compromising the benefits of a minimally invasive extraperitoneal repair.

## Conclusions

Lateral supravesical hernias are rare entities but should be considered when groin hernias present with atypical features. Intra-abdominal observation can be valuable in establishing a definitive diagnosis when imaging is inconclusive. Although the TEP approach is often avoided due to challenges in anatomical orientation, it may reduce the risk of organ injury, and its combination with intra-abdominal visualization could be considered a safe and effective strategy in these uncommon cases.

## References

[1] Yasukawa D, Aisu Y, Hori T (2020). Crucial anatomy and technical cues for laparoscopic transabdominal preperitoneal repair: advanced manipulation for groin hernias in adults. World J Gastrointest Surg.

[2] Cooper A (1844). The Anatomy and Surgical Treatment of Inguinal And Congenital Hernia. https://europepmc.org/backend/ptpmcrender.fcgi?accid=PMC5761448&blobtype=pdf.

[3] Lee SR (2017). Clinical characteristics and laparoscopic treatment of supravesical hernia. J Laparoendosc Adv Surg Tech A.

[4] Katsaros I, Routsi E, Papapanou M (2020). Supravesical hernias: a systematic review of the literature. ANZ J Surg.

[5] Bouassida M, Sassi S, Touinsi H (2012). Internal supravesical hernia - a rare cause of intestinal obstruction: report of two cases. Pan Afr Med J.

[6] Shibuya N, Ishizuka M, Iwasaki Y, Takagi K, Nagata H, Aoki T, Kubota K (2017). Usefulness of a laparoscopic approach for treatment of small-bowel obstruction due to intersigmoid hernia: a case report. Surg Case Rep.

[7] Fujimoto G, Deguchi T, Shirai J (2024). Transabdominal preperitoneal repair for an external supravesical hernia with an incarcerated ovary: a case report. Cureus.

[8] Elyamine O, Bensardi F, Majd A, Abdelilah EB, Mounir B, Khalid EH, Abdelaziz F (2021). Strangulated internal supravesical hernia associated with left inguinal hernia: a very rare case report of acute intestinal obstruction. Ann Med Surg (Lond).

[9] Bittner R, Bingener-Casey J, Dietz U (2014). Guidelines for laparoscopic treatment of ventral and incisional abdominal wall hernias (International Endohernia Society (IEHS)-part 1. Surg Endosc.

[10] Miserez M, Alexandre JH, Campanelli G (2007). The European Hernia Society groin hernia classification: simple and easy to remember. Hernia.

[11] Kenji A, Jun K, Yuki N, Daisaku U, Teruhiko W, Masahiko O (2019). A case of laparoscopic hernia repair for an external supravesical hernia: a case report (Article in Japanese). Isho.

[12] Asanuma K, Yoshida M, Takanashi S, Kashiyama M, Ishigo-Oka M, Kawashima H (2013). A case of internal supravesical hernia repaired by laparoscopic surgery (Article in Japanese). Nihon Geka Gakkai Zasshi.

[13] Kameyama T, Yahagi M, Masuda Y, Takei T, Kameyama T, Akiyama Y (2022). A recurrent extraperitoneal bladder hernia successfully treated with the TEP approach: a case report (Article in Japanese). Jpn J Surg.

[14] Köckerling F, Bittner R, Kuthe A (2017). Laparo-endoscopic versus open recurrent inguinal hernia repair: should we follow the guidelines?. Surg Endosc.

[15] Singh AP, Hivre MD, Gupta DO, Rokade AA (2025). A paradigm shift in the reporting and treatment of supravesical hernias with laparoscopic hernia repair: a case report. World J Laparosc Surg.

